# Predictors of Neonatal Sepsis in Hospitals at Wolaita Sodo Town, Southern Ethiopia: Institution-Based Unmatched Case-Control Study, 2019

**DOI:** 10.1155/2020/3709672

**Published:** 2020-10-30

**Authors:** Atkuregn Alemayehu, Mihiretu Alemayehu, Aseb Arba, Hanna Abebe, Abraham Goa, Kebreab Paulos, Mohammed Suleiman Obsa

**Affiliations:** ^1^Wolaita Sodo University, College of Medicine and Health Sciences, Institutional Quality Assurance, Ethiopia; ^2^Wolaita Sodo University, College of Medicine and Health Sciences, School of Public Health, Ethiopia; ^3^Wolaita Sodo University, College of Medicine and Health Sciences, School of Nursing, Ethiopia; ^4^Wolaita Sodo University, College of Medicine and Health Sciences, School of Midwifery, Ethiopia; ^5^Wolaita Sodo University, College of Medicine and Health Sciences, School of Anesthesia, Ethiopia

## Abstract

**Background:**

Neonatal sepsis plays a significant role in neonates' mortality in developing countries accounting for 30-50% of total deaths each year. Gaining insight into neonatal sepsis predictors will provide an opportunity for the stakeholders to reduce the causes of neonatal sepsis. This research is aimed at determining the predictors of neonatal sepsis at Wolaita Sodo University Teaching Referral Hospital and Sodo Christian General Hospital, Ethiopia, April-July 2019.

**Method:**

This study employed an institution-based unmatched case-control study by selecting neonates in selected hospitals through consecutive sampling technique. The cases of this study are neonates diagnosed with sepsis. The study used a pretested structured questionnaire for a face-to-face interview to collect data from index mothers. Besides, the review of the record was done using checklists. The data were entered into EpiData version 3.1 and exported to Statistical Package for the Social Sciences version 24.0 for analysis. The study used descriptive, bivariate, and multivariate analyses. The odds ratio with 95% confidence interval was used to measure the association's strength. *p* < 0.05 was the cut-off point for declaration of statistical significance for the multivariate analysis.

**Results:**

Factors significantly associated with neonatal sepsis among neonates were maternal age of 15-20 years and 21-30 years, mothers with low income/wealth, history of urinary tract infections/sexually transmitted infections, presence of intrapartum infections, antenatal care follow‐up < 3 visits, Apgar (Appearance, Pulse, Grimace, Activity, and Respiration) score < 7, low birth weight, and the time in which breastfeeding started after delivery < 60 minutes.

**Conclusion:**

Maternal age, wealth/income, maternal urinary tract infections/sexually transmitted infections, intrapartum fever, antenatal care visit ≤ 3 times, Apgar score < 7, low birth weight, and starting time of breastfeeding were independent predictors of neonatal sepsis. Therefore, maternal health education during antenatal care visits, perinatal and newborn care, and early initiation of breastfeeding might decrease neonatal mortality and morbidity due to sepsis.

## 1. Introduction

Neonatal sepsis is a systemic inflammatory response syndrome in the presence of infection in a neonate. It may appear in an early stage, which occurs at the first 72 hours of life, and after this period, it is characterized as a late-onset, usually caused by contact with pathogens acquired after birth. An infection could be of bacterial, viral, fungal, or rickettsial origin [1]. Sepsis is the commonest cause of neonatal morbidity and mortality. It is responsible for about thirty up to fifty percent of total neonatal deaths in developing countries [2, 3].

Neonatal sepsis occurs during the first 28 days of life. It is estimated to cause twenty-six percent of all neonatal deaths worldwide [4]. In sub-Saharan Africa, 17% of neonatal deaths are due to neonatal sepsis [2]. A recent study from Ethiopia indicated that neonatal sepsis is the major newborn killer, accounting for more than one-third of neonatal deaths [5–9].

Strategies that can prevent and treat neonates with sepsis are very important to increase a newborn's survival progress. Moreover, epidemiological data from developing countries showed the presence of differences in risk, prevalence, affecting factors, and mortality compared to developed countries [2, 5–12].

In the study area, little was known regarding risk factors of neonatal sepsis. Identifying risk factors will help neonatal care providers provide a risk-based diagnosis of neonatal sepsis which is one of the common difficulties for the treatment of neonatal sepsis. Therefore, this study is aimed at determining neonatal sepsis predictors among neonates in hospitals at Wolaita Sodo town, Southern Ethiopia.

## 2. Objective

The objective is to determine predictors of neonatal sepsis among neonates in hospitals at Wolaita Sodo town, Wolaita Zone, Southern Ethiopia, April 15-July 15, 2019.

## 3. Methods

### 3.1. Study Setting

The study setting was WSUTRH and SCGH, located in Wolaita Sodo town, Wolaita Zone. WSUTRH is the only referral hospital in Wolaita Zone and also serves about 3 million people. NICU (Neonatal Intensive Care Unit) is under maternal, neonatal, and child health services. It is a separate unit that started service in December 2014, with kangaroo mother care which has four beds, mothers waiting in the area or septic room with 16 beds, and a hot room with ten beds, and in total, there are 30 beds in NICU. Three pediatricians, four obstetricians, one gynecologist, and twelve neonatal specialized nurses provide the service in WSUTRH.

The SCGH is the only private general hospital in Sodo providing orthopedic and general, maternity, NICU, and pediatric services. Maternal, neonatal, and child health services in SCGH include NICU with kangaroo mother care, mothers' waiting room/septic room, full equipment, labor and delivery rooms, PNC (Postnatal Care), gynecology ward, and ANC unit.

### 3.2. Study Design and Period

This study employed an institution-based unmatched case-control study design in WSUTRH and SCGH from April to July, 2019.

### 3.3. Population

The source population is composed of all neonates of mothers who got MNCH (NICU, PNC, labor and delivery, and postanesthesia care unit) services in two hospitals. All selected neonates (during the study period) from the two hospitals were the study population of the study.

### 3.4. Inclusion and Exclusion Criteria

The study's cases were all neonates who developed a pediatrician-confirmed sepsis in the two hospitals at the time of data collection. The controls were all neonates who did not develop sepsis at the time of data collection as confirmed by the pediatrician during the study period.

The exclusion criteria for the study were neonates without their mother and neonates with an incomplete chart such as missing identification, diagnosis, and result. Besides, mothers with hearing or speaking disabilities were also excluded from the study.

### 3.5. Sample Size and Sampling Technique

A double population formula, using OpenEpi version 2, was used to determine the sample size of the study. The assumptions were the following: one to four ratio of the case to the control (1 : 4), 95% level of confidence, 90% power, and 10% nonresponse rate. Accordingly, the total sample size was 385 (77 cases and 308 controls).

### 3.6. Sampling Procedure

Cases have been selected consecutively from mothers of neonates attending maternal, neonatal, and child health services in WSUTRH and SCGH. The next immediate four corresponding controls have been selected by a consecutive method on the same day and in the same unit.

### 3.7. Study Variables

The dependent variable is neonatal sepsis.

The independent variables are the following: maternal sociodemographic variables (maternal age, maternal religion, maternal educational, maternal occupation, maternal marital status, occupational status of the husband, educational status of the husband, residence, and wealth), maternal obstetric variables (prolonged rupture of membrane (PROM), foul-smelling vaginal discharge/fluid, history of UTI/STI, APH, parity, intrapartum infection, and ANC visit), neonatal variables (age of neonate in days, sex of neonate, birth weight, congenital anomalies, birth trauma, birth asphyxia, Apgar score, gestational age (g.a.), and immediate cry), health facility and related factors (place of delivery, length of health facility, and common means of transportation), maternal and newborn clinical care, and related factors (invasive procedures, mode of delivery, attendant during delivery, cord cut, cord care, and frequency of digital per-vaginal examination).

### 3.8. Data Collection Tools

A pretested interviewer-administered questionnaire and checklists were the data collection tools of the study. The questionnaire was developed by reviewing different kinds of literature and checklists of WHO's Integrated Management of Neonatal and Childhood Illness (IMNCI), possible serious infection (PSI), Young Infants Clinical Sign Study (YICSS), and other relevant pieces of literature. Besides, questions were adapted from tools used in other studies to investigate risk factors for neonatal sepsis [2, 13, 14]. Initially, it was designed in English and translated to Amharic and back to English to check the questionnaire's consistency and appropriateness.

The questionnaire inquires about maternal sociodemographic characteristics, maternal obstetric factors, neonatal factors, health facility and related factors, maternal and newborn clinical care (MNCH), and related factors.

### 3.9. Data Collectors

Six trained bachelor nurses specialized in neonatal care collected the data with the supervision of two senior nurses who have previous experience in the supervision of data collection.

### 3.10. Data Collection Procedure and Quality Assurance

Data was collected through face-to-face interviews of the mother, review of the mother's chart, registration book records in MNCH service, record review of laboratory results, and chart view of the index mothers. Two days of training was given to data collectors to familiarize the data collectors with tools and procedures. The necessary adjustments were then made after doing a pretest on 4 cases and 16 controls in Dubbo Hospital. The supervisors have checked data from each respondent for its completeness, clarity, consistency, and accuracy. The principal investigator made continuous follow-up and supervision throughout the data collection period.

### 3.11. Operational Definition

*Neonate:* baby's age less than 28 days.

*Sepsis:* a life-threatening condition that arises when the body's response to an infection injures its tissues and organ (WHO).

*Neonatal sepsis:* when there was generalized infection with fever, Apgar score < 7, increased heart rate, increased breathing rate, and confusion documented within the first 28 days of life, confirmed by complete blood count results and by the pediatrician.

*Cases:* diagnosed by using the following: neonates with clinical signs of possible severe infection (PSI), according to the Young Infants Clinical Sign Study (YICSS), and criteria of WHO's Integrated Management of Neonatal and Childhood Illness (IMNCI) guidelines with hematologic criteria [15]. Another study included cyanosis and grunting [16]. The presence of any one of the seven clinical signs and symptoms predicts severe illness. This is based on an expert pediatrician's or physician's decision including other symptoms and signs such as bradycardia, tachycardia, irritability, oxygen requirement, and increased frequency of apnea [15].

*Controls:* neonates who did not fulfill sepsis criteria in two hospitals.

*Early neonatal sepsis:* sepsis presenting in the first 72 hours of life.

*Late neonatal sepsis:* presentation of sepsis after 72 hours of life.

*Postpartum infections:* infections that occurred from the immediate childbirth up to six weeks.

*Meconium-stained amniotic fluid (MSAF):* considered if the amniotic fluid was green/brown or mixed with meconium or appears meconium-stained on the baby (WHO).

*Prolonged rupture of membrane (PROM):* more than 18-hour delay in the period of “membrane” rupture to child delivery.

*Term newborn:* babies born after 37 completed weeks of gestation (WHO).

*Low birth weight:* a birth weight less than 2,500 grams at birth.

### 3.12. Data Processing and Analysis

The researchers checked for the completeness and consistencies of the data. Then, they cleaned, coded, and entered the data using EpiData version 3.1 and exported to SPSS version 24.0 statistical software for analysis. Descriptive statistics have been used to describe the study population concerning relevant variables by using frequencies. The wealth index was assessed by using household assets via principal component analysis (PCA). The variables with a *p* value of less than 0.25 and variables checked for multicollinearity in the bivariate analysis became a multivariate analysis candidate. Multivariate analysis was done to control for possible confounders using binary logistic regression to identify independent predictors of neonatal sepsis occurrence. *p* value and odds ratio (AOR) were used to measure the presence and strength of the association of variables with the occurrence of neonatal sepsis. A *p* value of less than 0.05 is a cut-off point for declaration of statistical significance of association with neonatal sepsis.

### 3.13. Ethical Considerations

The Ethical Review Board of Wolaita Sodo University, College of Health Science and Medicine, approved the study and provided a written letter of approval to collect data from mothers or caretakers aged 15 years and above on behalf of the neonate. The results of the study did not include the participant's identification. To ensure confidentiality, the collected data was used only for the intended purpose of the study. Informed written consent was obtained from mothers or caretakers of neonates to confirm willingness. Data collectors explained an outline of the purpose of the study to every mother or caretaker who agreed to participate in the study. Besides, the data collectors also informed mothers that they have the right to refuse or terminate at any point in the interview.

## 4. Result

### 4.1. Maternal Sociodemographic and Wealth Index Characteristic

This study involved a total of 385 neonates among which 77 had sepsis (cases) and 308 had no sepsis (controls), making a response rate of 100%. The mean (±SD) age of mothers was 26.37 ± 5.21 years ranging from 15 to 42 years. Thirty-four (44.1%) of cases and 116 (37.7%) of controls were living in rural areas. Regarding marital status, 72 (93.5%) of cases and 299 (97.1%) of controls were married. Thirty-two (41.5%) of cases and 82 (26.6%) of controls were housewives by their occupation, 8 (10.4%) of cases and 35 (11.4%) of controls of mothers had not attended formal education, and 11 (14.3%) of cases and 24 (07.8%) of controls of husbands of index mothers had not attended formal education. Twenty-eight (36.4%) of cases and 101 (32.8%) of controls were from a family with a low income as shown in Table 1.

### 4.2. Maternal Obstetric Factors of Neonatal Sepsis

249 (80.8%) of mothers of controls and 45 (58.4%) of cases had antenatal care (ANC) visit ≤ 3 times during the index pregnancy. Mothers of 19 (24.7%) of cases and 97 (31.5%) of controls were primigravida. The mothers who had a history of urinary tract infections or sexually transmitted infections (UTI/STI) during the index pregnancy in the cases were 26 (33.8%), and in the controls, there were 46 (14.9%). Mothers with intrapartum fever during the index pregnancy in the cases were 37 (48.0%), and in the controls, there were 63 (20.4%). The mothers who delivered after developing obstetric complications among the cases were 36 (46.7%), and among the controls, there were 117 (37.9.1%), and mothers of seven (9.1%) cases and 8 (2.6%) controls developed APH in the index pregnancy as shown in Table 2.

### 4.3. Neonatal Factors

Two hundred and one (84.7%) of controls and 49 (63.6%) of cases were born at term. 45 (58.4%) of cases' mothers and 277 (89.9%) of controls' mothers delivered their children with normal birth weight. 34 (44.1%) of cases and 26 (8.4%) of controls had an Apgar score of less than 7. Concerning the age of neonates, 76 (98.7%) of the cases and 305 (99.9%) controls were under the age of 7 days.

Among cases, 57 (74.1%) neonates started breastfeeding before 60 minutes after birth, while 202 (65.6%) of controls started before 60 minutes. Seventy-two (93.5%) of cases and 295 (95.8%) of controls had no history of trauma, which disrupts skin at birth. Similarly, 75 (97.4%) of cases and 306 (99.4%) of controls had no congenital abnormalities at birth, as shown in Table 3.

### 4.4. Health Facility and Related Factors

A total of two hundred seventy-five (89.6%) controls and 67 (87%) cases were born at the hospital. Sixty-nine (89.6%) of cases' and 295 (95.8%) of controls' mothers used vehicles to come to the health facility. Similarly, fifteen (19.5%) of cases and 142 (46.1%) of controls traveled more than 60 minutes to arrive at the health facility as shown in Table 4.

### 4.5. Maternal and Newborn Clinical Care-Related Factors

231 (75.3%) of controls' and 57 (74.0%) of cases' mothers delivered their neonates by spontaneous vaginal delivery. The delivery of three hundred five (99.0%) controls and 72 (93.5%) cases was attended by a health professional. The mothers who had a history of per-vaginal examination less than three times in the cases were 31 (40.2%), and in the controls, there were 50 (16.2%). In most deliveries, professionals used scissors to cut the cord of 64 (83.1%) cases and 301 (97.7%) of controls. Seventy-three (94.8%) of cases and 298 (96.8%) of controls had no history of peripherally inserted IV line. Only 1 (0.32%) of controls delivered through an invasive procedure, as shown in Table 5.

### 4.6. Bivariate and Multivariable Logistic Regression Analysis Result

In the bivariate analysis, maternal age, wealth index, parity, history of UTI/STI, intrapartum infection/fever, prolonged rupture of amniotic membrane, foul-smelling liquor/amniotic fluid, obstetric complication, ANC visit ≤ 3, bleeding disorder during the index pregnancy, starting time of breastfeeding, Apgar score < 7, low birth weight, per-vaginal examination, and common means of transportation have shown statistically significant association (*p* < 0.25) with neonatal sepsis. In the multivariate analysis, the number of maternal ANC visit, history of maternal UTI/STI, mothers with infection during labor, Apgar score < 7, low birth weight, mothers who started breastfeeding > 60 minutes, maternal age of 15-20 and 21-30, and maternal income/wealth level were found to be significantly associated factors of neonatal sepsis. Those mothers who had ANC visits ≤ 3 times were about three times more likely to have sepsis. History of UTI/STI on mothers in index pregnancy was significantly associated with the risk of neonatal sepsis. Mothers with infection during labor were nearly three times more likely to have neonatal sepsis. An Apgar score < 7 was found to be significantly associated with the risk of acquiring neonatal sepsis.

Neonates with low birth weight were significantly associated with the risk of occurrence of neonatal sepsis. Mothers who started breastfeeding > 60 minutes after delivery were around twelve times higher to develop neonatal sepsis. Maternal age of 15-20 years and 21-30 years was nearly four and five times highly exposed to developing neonatal sepsis. Last but not least, mothers with low income/wealth were nearly three times more likely to be exposed to develop neonatal sepsis as shown in Table 6.

## 5. Discussion

In this study, 98.7% of cases were with early-onset neonatal sepsis (<7 days), which is less compared with the studies conducted earlier in our country in Bishoftu and Gondar which was 81.4% and 81.8% [6, 9]. It might be due to an increase in the patient load and a high amount of referrals from catchment health facilities.

This study found that maternal age of 15-20 and 21-30 has 4 and 5 times higher odds of developing neonatal sepsis compared to neonates of mothers who were >31 years of age (AOR = 4.13, 95% CI [1.71, 9.93] and AOR = 4.96, 95% CI [1.25, 19.6]). This finding goes in line with the study conducted in Ghana, in which maternal age was a significant factor. Specifically, women aged from 21 to 30 years were 1.95 times less likely to have infants with neonatal sepsis compared to those aged less than 20 years [17]. The current study's finding goes in line with the study conducted in Pakistan which showed that the maternal age of 23 years (range of 18-36 years) was highly prone to develop neonatal sepsis [18]. This might be due to a lack of preconception care for women.

In this study, neonates who were born from mothers with low income/wealth have nearly 3 times higher odds of developing sepsis than mothers with high income/wealth (AOR = 2.76, 95% CI [1.02, 7.52]). This finding goes in line with the study conducted in Pakistan, which shows that each year almost one million newborns die from infections in households with low household income [19]. This might be due to lack of awareness, fear of affordability to health facilities, and mother's nutritional imbalance.

In this study, neonates born from mothers who had a history of UTI/STI during the index pregnancy have 3 times higher odds of developing sepsis than their counterparts (AOR = 2.72, 95% CI [1.06, 6.97]). This is consistent with the study findings conducted in Ghana, where neonates born from women with a UTI history were more likely to develop sepsis. This finding goes in line with the study conducted in Bishoftu, Ethiopia, which showed that maternal UTI/STI was a significant factor for the development of neonatal sepsis [12, 20]. This might be due to the fact that the wall of the birth canal in women with urinary tract infection is colonized with pathogens.

Neonates born to mothers who had a fever during labor have 3 times higher odds of developing sepsis than their counterparts (AOR = 2.93, 95% CI [1.32, 6.47]). This finding was going in line with studies conducted in Mekele, India, and Pakistan, where neonates delivered from women with intrapartum fever were more likely to develop sepsis than those without fever [2, 21, 22]. This might be because of the reason that fever is a sign of local or systemic infections such as chorioamnionitis and urinary tract infection, which are frequently transmitted to the baby in the uterus or during passage through the canal.

This study showed that the neonates delivered from the mother who had ANC visit ≤ 3 visits were three times at higher odds of developing sepsis compared to mothers who had 4 and above ANC visits (AOR = 2.94, 95% CI [1.21, 7.16]). This finding goes in line with the studies conducted in South Ethiopia, Uganda, and India, in which sick newborns (whose mothers had no ANC visit) were more likely to have sepsis compared to controls (OR = 3.21; 95% CI 1.24–8.33) [23–25]. This might be because of the reason that women who had full ANC visits might have a better understanding and medical care of risk factors, than those women with incomplete ANC visits. This indicates that having an ANC visit during pregnancy indirectly saves the lives of mothers and babies by promoting and establishing good health before childbirth and during the early postnatal period.

This study found that the neonates with Apgar score < 7 have fifteen times higher odds of developing sepsis compared to neonates with Apgar score > 7 (*AOR* = 15.1, 95% CI [4.78, 47.65]). This finding goes in line with the study conducted in Ghana and Mekele that found that the odds of developing neonatal sepsis were higher among neonates with Apgar score < 7 than neonates with Apgar score > 7. The Apgar score is the overall indicator for the newborn's state in the extrauterine environment, and neonates with a low Apgar score could be in a state of bradycardia asphyxia and need emergency support. Besides, the newborn could have acquired the pathogen vertically (mother to fetus) in the uterus before delivery, which results in sepsis [2, 18, 26–28].

The neonates with low birth weight have eight times higher odds of developing neonatal sepsis than those with birth weight greater than 2500 gm (AOR = 8.46, 95% CI [3.52, 20.3]) in this study. This finding was similar to the study conducted in Indonesia and Pakistan in which neonates who were delivered with low birth weight < 2500 gm were more likely to develop neonatal sepsis than neonates with birth weight > 2500 gms [22, 29]. This might be due to the reason that physical barriers such as skin, the mucosa membrane, and chemicals (antibacterial or those that inhibit the adhesion of pathogens to the host) begin to develop around 32-34 weeks of gestation and then become matured after the birth. Hence, the IgA level is produced by the mucosa protection layer, which is lower on the LBW.

Early initiation of breastfeeding had shown a significant effect on neonatal sepsis development in the current study. Neonates who started breastfeeding after 60 minutes of delivery have twelve times higher odds of developing sepsis than neonates who started breastfeeding within 60 minutes of delivery (AOR = 12.5, 95% CI [3.80, 41.7]). This finding was similar to the result of a study conducted in Uganda [23]. Similarly, the “New Approaches to Preventing, Diagnosing, and Treating Neonatal Sepsis” study in Pakistan showed that early initiation and exclusive breastfeeding account for less exposure to neonatal sepsis [30].

## 6. Conclusions

Finally, maternal age, wealth index, history of maternal UTI/STI, intrapartum fever, ANC visit ≤ 3, Apgar score < 7, low birth weight, and starting time of breastfeeding were identified as an independent predictor of neonatal sepsis. This study has also seen that neonatal sepsis was higher in the first week of life.

### 6.1. Recommendation

(1) Health institutions should
  - mobilize and support the development of the habit of early health-seeking behavior for health problems during pregnancy, like seeking care for UTI/STI and institutional delivery
  - encourage mothers to breastfeed their newborns and initiate as early as possible
  - enable mothers to adhere to focused ANC visit according to the schedule
(2) Governmental bodies
  - should support mothers with low income by using different strategies, like a small package with stakeholders, to support their household economy
  - should address all mothers through different strategies to support them to complete the ANC visit as recommended
  - should launch preconception care for women in reproductive age groups
(3) Health professionals
  - should give health education to each mother when attending ANC clinic, to continue visit according to FMOH recommendation, and inform mothers on different topics like early ANC visit, maternal nutrition, hospital delivery, early initiation of breastfeeding and tracing defaulters of ANC visit, the sign of UTI/STI, and intrapartum fever/infection
  - should pay attention to a mother giving birth to detect fever and other complications
  - should follow labor based on a partograph to detect and prevent complications that may cause a low Apgar score on the newborn

## Figures and Tables

**Table 1:** Maternal sociodemographic and wealth factor for neonatal sepsis among neonates at SCGH and WSUTRH, Wolaita Sodo town, 2019.

Variables	Categories	Cases, *n* = 77 (%)	Controls, *n* = 308 (%)	Chi square
Maternal age	15-20	9 (11.7)	52 (16.9)	
	21-30	42 (54.5)	218 (70.8)	0.001
	>31	26 (33.8)	38 (12.3)	

Marital status	Married	72 (93.5)	299 (97.1)	
	Single	4 (5.2)	4 (1.3)	
	Divorced	0 (0)	2 (0.6)	0.162
	Widowed	1 (01.3)	3 (1)	

Residence	Urban	43 (55.8)	192 (62.3)	0.296
	Rural	34 (44.1)	116 (37.7)	

Maternal education	No formal education	8 (10.4)	35 (11.4)	
	Primary school	23 (29.9)	116 (37.7)	0.300
	Secondary school	33 (45.8)	97 (31.5)	
	Diploma and above	13 (16.9)	60 (19.5)	

Maternal occupation	Housewife	32 (41.5)	82 (26.6)	
	Farmer	7 (9.1)	14 (4.5)	
	Daily laborer	4 (05.2)	16 (5.2)	
	Student	4 (5.2)	63 (20.4)	
	Governmental employee	8 (10.4)	56 (18.2)	0.007
	Private business\merchant	16 (20.8)	55 (17.8)	
	NGO employee	6 (7.8)	22 (7.1)	

Husband education	No formal education	11 (14.3)	24 (7.8)	
	Primary school	14 (18.2)	67 (21.8)	
	Secondary school	17 (22.1)	79 (25.7)	0.034
	Diploma and above	31 (40.2)	131 (42.5)	

Wealth index	Low	28 (36.4)	101 (32.8)	
	Middle	35 (45.4)	93 (30.2)	0.004
	High	14 (18.2)	114 (37.0)	

**Table 2:** Maternal obstetric factors of neonatal sepsis among neonates at SCGH and WSUTRH, Wolaita Sodo town, 2019.

Variables	Categories	Cases (77)	Controls (308)	Chi square
Parity	Primigravida	19 (24.7)	97 (31.5)	0.243
	Multiparous	58 (75.3)	211 (68.5)	

ANC visit recommended	≥4 visits	32 (41.5)	59 (19.1)	0.001
	≤3 visits	45 (58.4)	249 (80.8)	

Intrapartum infection/fever	Present	37 (48.0)	63 (20.4)	0.001
	Not present	40 (52.0)	245 (79.5)	

UTI/STI	Present	26 (33.8)	46 (14.9)	0.001
	Not present	51 (66.2)	262 (85.1)	

PROM	Present	32 (41.5)	39 (12.7)	0.001
	Not present	45 (58.4)	269 (87.4)	

Foul-smelling liquor	Present	18 (22.1)	18 (5.8)	0.001
	Not present	59 (76.6)	290 (94.1)	

Obstetric complication	Present	36 (46.7)	117 (37.9)	0.001
	Not present	41 (53.3)	191 (62.1)	

Bleeding disorder (APH)	Present	7 (9.1)	8 (2.6)	0.001
	Not present	70 (90.1)	300 (97.4)	

**Table 3:** Neonatal factors of neonatal sepsis among neonates at SCGH and WSUTRH, Wolaita Sodo town, 2019.

Variables	Categories	Cases (77)	Controls (308)	Chi square
Age of the neonate	<7 days	76 (98.7)	305 (99.0)	0.802
	8-28 days	1 (1.3)	3 (1.0)	

Sex of newborn	Male	49 (63.6)	182 (59.1)	0.466
	Female	28 (36.4)	126 (40.9)	

Gestational age of newborn in weeks	<34 WK	6 (07.8)	14 (4.5)	0.001
	34-37 WK	22 (28.6)	33 (10.7)	
	37-42 WK	49 (63.6)	261 (84.7)	

Birth weight	<2500 gm	32 (41.5)	31 (10.1)	0.001
	>2500 gm	45 (58.4)	277 (89.9)	

Congenital abnormalities	Yes	2 (2.6)	2 (0.6)	0.132
	No	75 (97.4)	306 (99.4)	

Trauma that disrupts skin at birth	Yes	5 (06.9)	13 (4.2)	
	No	72 (93.5)	295 (95.8)	0.398

Apgar score	<7	34 (44.1)	26 (8.4)	0.001
	7-10	43 (55.8)	282 (91.5)	

Start breastfeeding	>60 minutes	20 (25.9)	106 (34.4)	0.006
	<60 minutes	57 (74.1)	202 (65.6)	

**Table 4:** Health facility and related factor of neonatal sepsis among neonates at SCGH and WSUTRH, Wolaita Sodo town, 2019.

Variables	Categories	Cases (77)	Controls (308)	Chi square
Place of delivery	Hospital	67 (87.0)	275 (89.3)	
	Health centre	5 (6.5)	28 (10.0)	
	Private health facility	1 (1.3)	2 (0.65)	0.075
	Home	4 (5.2)	3 (0.97)	

Common means of transportation	On foot	8 (10.4)	13 (04.2)	0.033
	Vehicle	69 (89.6)	295 (95.8)	

Duration it takes from home to health facility	>60 minutes	15 (19.5)	142 (46.1)	
	45-60 minutes	25 (32.5)	68 (22.1)	
	30-45 minutes	23 (29.9)	67 (21.7)	0.001
	<30 minutes	14 (18.2)	31 (10.1)	

**Table 5:** Maternal and newborn clinical care-related factors of neonatal sepsis among neonates at SCGH and WSUTRH, Wolaita Sodo town, 2019.

Variables	Categories	Cases (77)	Controls (308)	Chi square
Mode of delivery	Spontaneous vaginal delivery	57 (74.0)	232 (75.3)	0.444
	Instrumental delivery	6 (7.8)	12 (3.9)	
	Caesarean section	13 (16.9)	62 (20.1)	
	Assisted breech/extraction	1 (01.3)	2 (0.65)	

Attendant during delivery	Traditional birth attendants	5 (6.5)	3 (0.97)	0.002
	Health professional	72 (93.5)	305 (99.0)	

Per-vaginal examination	<3 times	31 (40.2)	50 (16.2)	0.001
	4-6 times	30 (39.0)	152 (49.3)	
	>7 times	16 (20.8)	106 (34.4)	

Cord cut	Scissor	64 (83.1)	301 (97.7)	0.001
	New blade	6 (7.8)	7 (2.3)	
	Old blade	2 (2.6)	0 (0)	
	Do not know	5 (06.5)	0 (0)	

Cord care/clean	Yes	14 (18.2)	57 (18.5)	0.732
	No	9 (11.7)	27 (8.8)	
	Do not know	54 (70.1)	224 (72.7)	

Peripherally inserted IV line	Yes	4 (5.2)	10 (3.2)	0.414
	No	73 (94.8)	298 (96.8)	

IV insertion site	Central catheter line	2 (02.6)	0 (0)	0.050
	Umbilical line	0 (0)	1 (0.32)	
	Central intravenous line	2 (2.6)	9 (2.9)	

Invasive procedure	Yes	0 (0)	1 (0.32)	0.617
	No	77 (100)	307 (99.7)	

**Table 6:** Bivariate and multivariate analyses of neonatal sepsis neonates at SCGH and WSUTRH, Wolaita Sodo town, 2019.

Variables	Categories	Cases (77)	Controls (308)	COR [95% CI]	AOR [95% CI]
Maternal age	15-20	9 (11.7)	52 (16.9)	0.25 [0.10, 0.60]	4.13 [1.71, 9.93]^∗^
	21-30	42 (54.5)	218 (70.8)	0.89 [0.41, 1.96]	4.96 [1.25, 19.6]^∗^
	>31	26 (33.8)	38 (12.3)	1	1

Wealth index	Low	28 (36.4)	101 (32.8)	2.25 [1.13, 4.52]	2.76 [1.02, 7.52]^∗^
	Middle	35 (45.4)	93 (30.2)	0.73 [0.41, 1.30]	0.79 [0.32, 1.99]
	High	14 (18.2)	114 (37.0)	1	1

Parity	Primigravida	19 (24.7)	97 (31.5)	0.71 [0.40, 1.62]	0.71 [0.28, 1.78]
	Multiparous	58 (75.3)	211 (68.5)	1	1
Obstetric complication	Yes	36 (46.7)	117 (37.9)	1.43 [0.87, 2.37]	1.71 [0.77, 3.79]
	No	41 (53.3)	191 (62.1)	1	1

Intrapartum fever	Yes	37 (48.0)	63 (20.4)	3.59 [2.12, 6.08]	2.93 [1.32, 6.47]^∗^
	No	40 (52.0)	245 (79.5)	1	1

UTI/STI	Yes	26 (33.8)	46 (14.9)	2.90 [1.64, 5.11]	2.72 [1.06, 6.97]^∗^
	No	51 (66.2)	262 (85.1)	1	1

PROM	Yes	32 (41.5)	39 (12.7)	4.90 [2.79, 8.62]	1.50 [0.55, 4.05]
	No	45 (58.4)	269 (87.4)	1	1

Foul-smelling liquor	Yes	18 (22.1)	18 (5.8)	4.91 [2.41, 10.0]	2.42 [0.70, 8.32]
	No	59 (76.6)	290 (94.1)	1	1

No. of ANC visit	3 & below visit	32 (41.5)	59 (19.1)	3.00 [1.75, 5.12]	2.94 [1.21, 7.16]^∗^
	4 & above visit	45 (58.4)	249 (80.8)	1	1

Bleeding disorder (APH)	Yes	7 (9.1)	8 (2.6)	3.75 [1.31, 10.68]	2.13 [0.46, 9.80]
	No	70 (90.1)	300 (97.4)	1	1

Apgar score	<7	34 (44.1)	26 (08.4)	8.57 [4.69, 15.67]	15.1 [4.78, 47.6]^∗^
	7-10	43 (55.8)	282 (91.5)	1	1

Birth weight	<2500 gm	32 (41.5)	31 (10.1)	6.35 [3.53, 11.41]	8.46 [3.52, 20.3]^∗^
	>2500 gm	45 (58.4)	277 (89.9)	1	1

Start of breastfeeding time	>60 minutes	20 (25.9)	106 (34.4)	0.69 [0.38, 1.59]	12.5 [3.80, 41.7]^∗^
	<60 minutes	57 (74.1)	202 (65.6)	1	1

Common means of transportation	On foot	8 (10.4)	13 (4.2)	2.63 [1.05, 6.59]	2.14 [0.59, 7.76]
	Vehicle	69 (89.6)	295 (95.8)	1	1

Per-vaginal examination	>7 times	16 (20.8)	106 (34.4)	0.24 [0.12, 0.48]	1.36 [0.57, 3.25]
	4-6 times	30 (39.0)	152 (49.3)	0.76 [0.39, 1.47]	0.58 [0.22, 1.58]
	<3 times	31 (40.2)	50 (16.2)	1	1

NB: ^∗^significant at *p* < 0.05. COR: crude odds ratio; AOR: adjusted odds ratio.

## Data Availability

All data were made available within the manuscript.

## Abbreviations

ANC: Antenatal care
Apgar: Appearance, Pulse, Grammies, Activity, and Respiration
AOR: Adjusted odds ratio
CI: Confidence interval
COR: Crude odds ratio
IMNCI: Integrated Management of Neonatal and Childhood Illness
NICU: Neonatal Intensive Care Unit
PNC: Postnatal Care
PROM: Prolonged rupture of membrane
SCGH: Sodo Christian General Hospital
UTI: Urinary tract infection
WSUTRH: Wolaita Sodo University Teaching Referral Hospital.
